# Production and delivery of *Helicobacter pylori* NapA in *Lactococcus lactis* and its protective efficacy and immune modulatory activity

**DOI:** 10.1038/s41598-018-24879-x

**Published:** 2018-04-24

**Authors:** Xiaoyan Peng, Rongguang Zhang, Guangcai Duan, Chen Wang, Nan Sun, Linghan Zhang, Shuaiyin Chen, Qingtang Fan, Yuanlin Xi

**Affiliations:** 1 Department of Epidemiology and Statistics, College of Public Health, Zhengzhou University, Zhengzhou, 450001 China; 2 Henan Innovation Center of Molecular Diagnosis and Laboratory Medicine, Xinxiang Medical University, Xinxiang, 453003 China; 3 Department of Clinical Medicine, Zhengzhou University, Zhengzhou, 450001 China

## Abstract

*Helicobacter pylori* neutrophil-activating protein A subunit (NapA) has been identified as a virulence factor, a protective antigen and a potent immunomodulator. NapA shows unique application potentials for anti-*H. pylori* vaccines and treatment strategies of certain allergic diseases and carcinomas. However, appropriate production and utilization modes of NapA still remain uncertain to date. This work has established a novel efficient production and utilization mode of NapA by using *L. lactis* as an expression host and delivery vector, and demonstrated immune protective efficacy and immune modulatory activity of the engineered *L. lactis* by oral vaccination of mice. It was observed for the first time that *H. pylori* NapA promotes both polarized Th17 and Th1 responses, which may greatly affect the clinical application of NapA. This report offers a promising anti-*H. pylori* oral vaccine candidate and a potent mucosal immune modulatory agent. Meanwhile, it uncovers a way to produce and deliver the oral vaccine and immunomodulator by fermentation of food like milk, which might have striking effects on control of *H. pylori* infection, gastrointestinal cancers, and Th2 bias allergic diseases, including many food allergies.

## Introduction

*Helicobacter pylori* infects over half of the global population, causing a variety of serious diseases including gastric carcinomas^1^. The infection rates were more than 50.8% in developing countries while over 34.7% in developed countries^2^. Current therapies, such as triple therapies, quintuple therapies and high-dose dual therapies, are based on using bismuth, proton pump inhibitors and antibiotics. These therapies are facing challenge of increasing resistance to the first-line antibiotics like clarithromycin, metronidazole and levofloxacin^3–5^. Vaccination has been proposed as the most promising strategy for control of this infection, but so far no commercial vaccine is available^6^.

In two days post challenge with *H. pylori*, as an innate immune response, infiltration of macrophages and neutrophils occurs in gastric glandular tissues^7^. By three weeks after infection, an adaptive immune response has arisen, and a large amount of T lymphocytes, besides macrophages and neutrophils, infiltrate into gastric submucosa and mucosa^7^. In an acute *H. pylori* infection, the immune response in mice is characterized by Th1 and Th17 activity, while in a chronic infection, it is marked by mixed Th1/Th2 activity^7,8^. Most of the infected individuals have no or little manifestation but carry this bacterium all their lives. Accumulated evidences support that anti-*H. pylori* protective immunity induced by vaccination is predominantly attributed to enhanced Th1 and especially Th17 responses^9,10^.

*H. pylori* neutrophil-activating protein A subunit (NapA) was originally identified as a virulence factor for its ability to mediate binding of *H. pylori* to gastric mucus, attract and activate neutrophils, and promote gastric inflammation^11^. Recently, the immune modulatory activity and potential applications of NapA have been investigated. NapA, as a Toll-like receptor-2 (TLR2) agonist, can activate dendritic cells (DCs), eliciting high IL-12 and low IL-10 secretion^12,13^. Stimulation of human neutrophils and monocytes with NapA can induce expression of IL-12 and IL-23, and thereby shift antigen-specific T cell responses from a Th2 to a Th1 phenotype, which is characterized by high levels of interferon-gamma (IFN-γ) and tumor necrosis factor-alpha (TNF-α) production^13^. NapA can also depress the Th2 response by activation of Treg cells^14^. These findings suggest that NapA might be a new tool for future preventive and therapeutic strategies aimed at redirecting Th2 to Th1 responses, for instance, in vaccinology, allergy and cancer immunotherapy^13^.

NapA plays dual roles in dealing with oxidative stress^15^. While NapA mediates damage to DNA by stimulating neutrophil to produce reactive oxide species, it protects DNA by combating oxidative stress with its ferroxidase center^15,16^. NapA neither has toxic effect on monocytes and neutrophils nor reduces their viability or lifespan, although it can enhance production of nitric oxide^17,18^. These data warrant approaches on application of NapA as a vaccine candidate or immunotherapy agent^17^.

In vaccine formulation, mucosal vaccination followed by systemic immunization with NapA significantly enhanced specific local and systemic immune responses^19^. NapA, used in combination with mucosal adjuvant or delivered by attenuated pathogens, can induce remarkable protection against *H. pylori* challenges by oral vaccination^11,20^. Although the immune efficacy is compromised when using only NapA in immunization, this protein is still considered as a major antigen candidate for anti-*H. pylori* vaccines^11,20^.

As reported, NapA has considerable efficacy on alleviating Th2-based allergic diseases like asthma^21,22^. NapA can drive Th1 inflammation and inhibit Th2 responses in allergic bronchial asthma. Both systemic and mucosal administrations of NapA are capable of reducing eosinophils, immunoglobulin E (IgE) and Th2 cytokines in bronchi^22,23^. Current evidences support NapA to be a novel treatment strategy for allergic diseases^22,23^.

Additionally, NapA has been used in treatment for many malignant tumors, such as bladder cancer, breast cancer, hepatoma and neuroendocrine tumor in animal models^24–27^. As observed, local administration of NapA induces bladder tumor necrosis by activating cytotoxic Th1 response^26^. Engineering oncolytic viruses like measles virus and adenovirus to express NapA can significantly enhance their antitumor activity^27^. The therapeutic effects of NapA have been attributed to its role in maturation of DCs, attraction of immune cells, polarized activation of antigen specific T cells, and induction of pro-inflammatory cytokine release^13,24,28^.

Currently, NapA is produced and applied mainly by purification from the products of recombinant *E. coli* strains, or by expression and delivery in attenuated pathogens like *Salmonella typhimurium* and measles virus^29–31^. The present problems include the dependence on unsafe mucosal adjuvant for the purified NapA to take protective effect, the purification productivity, the effect of purification on the bioactivity, and safety of the attenuated pathogens as delivery vectors for human use^29–31^. For further utilization of NapA, it is critical to exploit novel ways for production and delivery of this crucial protein.

In contrast to the attenuated pathogens, *L. lactis* NZ3900 is a food-grade probiotic bacterium. Moreover, *L. lactis* dose not colonize the digestive tract, and thus less probably leads to tolerance towards the delivered immunogens, compared with human commensal bacteria^32^. To date, studies have used *L. lactis* to produce *H. pylori* antigens like UreB, Cag7 and NapA, resulting in immune efficacy from no protection to reduced bacterial colonization in mice^33–35^. Besides, certain *L. lactis* strains isolated from the environment were proved capable of promoting Th1 bias immune response, and show allergy-protective in mice^36,37^. So far, the efficacy of production and delivery of NapA in *L. lactis* remains uncertain.

Here, an engineered *L. lactis* strain expressing NapA was genetically constructed and used for oral vaccination of mice, and thereby the protection against *H. pylori* challenges and immune modulation of mice were evaluated. This paper offers a novel efficient production and utilization mode of NapA, a promising oral vaccine candidate and a potent mucosal immunomodulator.

## Results

### Engineered *L. lactis* comprising *napA*

**Figure 1:**
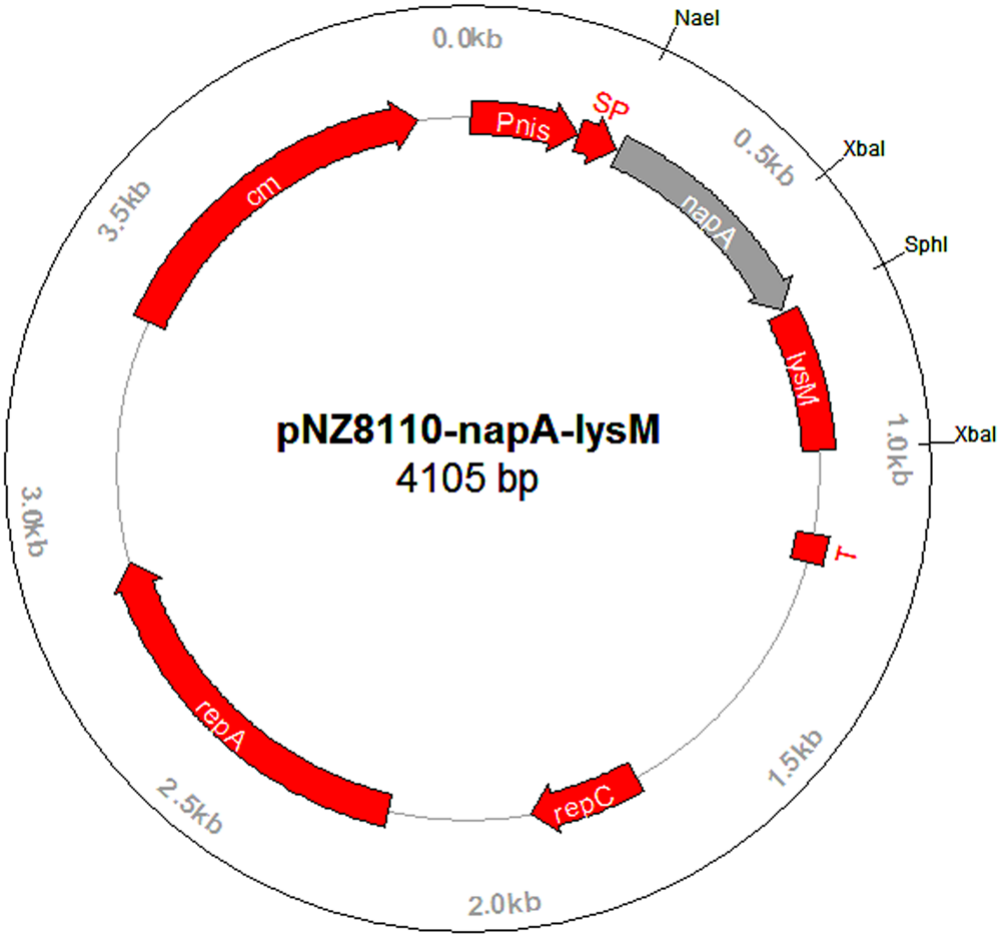
The schematic maps of the recombinant plasmid pNZ8110-*napA-lysM*. Pnis, nisA promoter; SP, signal sequence of *usp*45 gene; napA, *napA* gene coding region; T, terminator; *repA* and *repC*, replication gene A and C; cm, chloramphenicol resistance gene; *lysM*, the anchor motif of *L. lactis acmA* gene.

Gene sequencing showed that the sequence of the amplified *napA* gene was 432 bp in length (Supplementary Fig. 1) and identical to the published sequence (Genbank No. AY366361). It was confirmed by restrictive enzyme digestion and gene sequencing that the *napA* gene was exactly cloned in *L. lactis*, downstream of the nisin controlled promoter (*Pnis*) within the expression vector pNZ8110-*lysM*, generating pNZ8110-*napA*-*lysM* (Genbank No. KY385374). The schematic map of pNZ8110*-napA*-*lysM* is shown in Fig. 1.

### Expression and immunoreactivity of NapA

**Figure 2:**
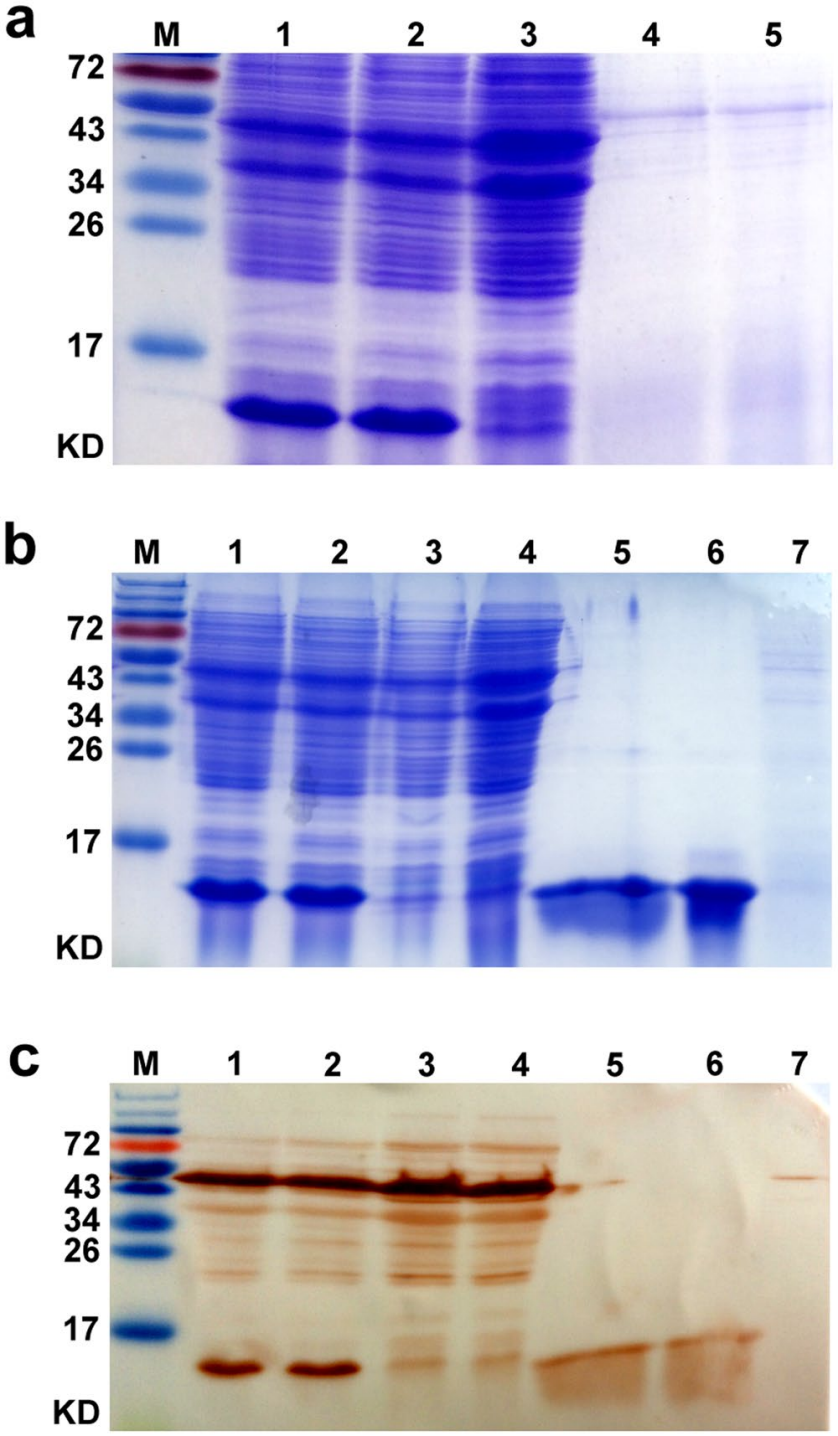
SDS-PAGE (**a**,**b**) and western blotting analysis (**c**) of *L. lactis* cell lysate, cell wall and cutrure supernatant proteins. The *L. lactis* strains were induced to express NapA using nisin. M, Protein markers. (**a**) Lane 1, 2, Cell lysates of *L. lactis* NZ3900 (pNZ8110-*napA-lysM*); Lane 3, Cell lysates of NZ3900 (pNZ8110*-lysM)*; Lane 4, 5, Culture supernatant of NZ3900 (pNZ8110-*napA*-*lysM*) and NZ3900 (pNZ8110*-lysM*), respectively. (**b**) Lane 1, 2, Cell lysates of NZ3900 (pNZ8110-*napA-lysM*); Lane 3, 4, Cell lysates of NZ3900 (pNZ8110*-lysM*); Lane 5, 6, Cell wall proteins of NZ3900 (pNZ8110-*napA*-*lysM*); Lane 7, Cell wall proteins of NZ3900 (pNZ8110*-lysM*). (**c**) Lane 1, 2, Cell lysates of NZ3900 (pNZ8110-*napA-lysM*); Lane 3, 4, Cell lysates of NZ3900 (pNZ8110*-lysM*); Lane 5, 6, Cell wall proteins of NZ3900 (pNZ8110- *napA*-*lysM*); Lane 7, Cell wall proteins of NZ3900 (pNZ8110*-lysM*). (**a**–**c**) were cropped from Supplementary Fig. 2a–c. SDS-PAGE and westernblot assays showed that the recombinant NapA protein was detectable both in cell lysate and cell wall protein samples, and possessed immunoreactivity with mouse anti-*H. pylori* sera.

The expression of NapA in engineered *L. lactis* was induced with nisin. SDS-PAGE assays of the cell lysates resulted in that a predominant band was present at approximately 14 kD position in the cell lysate and the cell wall protein samples of the engineered strain, while absent at the corresponding positions in the controls (Fig. 2a). No remarkable difference was detected in culture supernatant samples between the engineered strain and the control (Fig. 2b). The expressed NapA constitutes 15% of the cell lysate proteins of the engineered *L. lactis*. Western blotting assays showed that the 14 kD protein expressed in the engineered *L. lactis* yielded positive immunoreaction with the mouse anti-*H. pylori* sera (Fig. 2c). Figure 2a–c were cropped from Supplementary Fig. 2a–c. These findings demonstrated efficient expression of NapA in the engineered *L. lactis* and the antigenicity of the recombinant NapA.

### Immunization of mice and antibody assays

**Figure 3:**
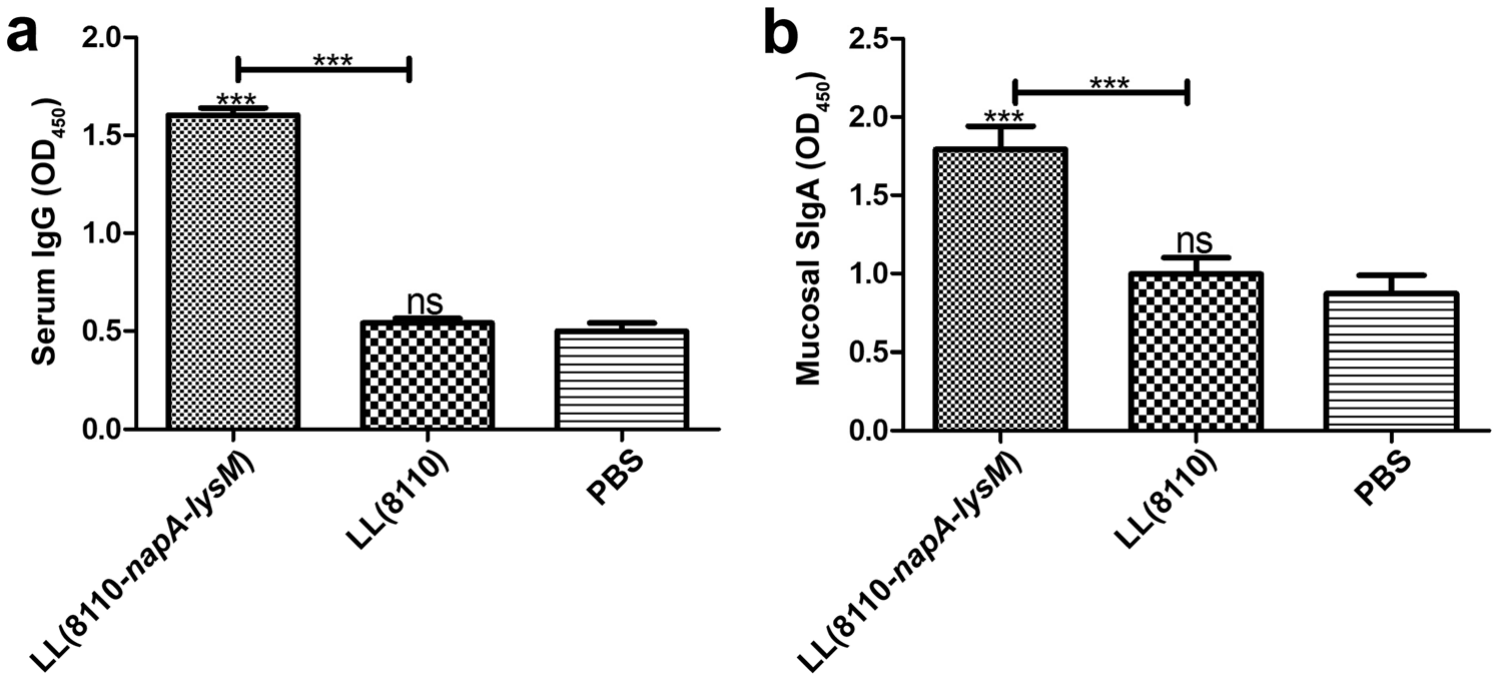
ELISA tests for NapA-specific serum IgG (**a**) and fecal SIgA (**b**) levels. The blood and feces of mice was sampled one week after the last oral immunization. The sera (1:50 diluted) and fecal samples were tested for NapA-specific antibodies. ns, no significance; ^*^P < 0.05; ^**^P < 0.01; ^***^P < 0.001, compared with the PBS or designated group. This figure showed that oral immunization with the engineered *L. lactis* expressing NapA evoked significantly elevated fecal SIgA and serum IgG responses in mice.

The NapA was purified from *E. coli* TB1 (pMAL-c2x-linker-*napA*), resulting in product with a purity of estimated 90%. The purified NapA (4 μg/mL) was used to coat ELISA microplates. The sera (1:50 diluted) and fecal samples were tested by ELISA for NapA-specific antibodies. As results, both fecal SIgA and serum IgG levels were higher in the LL(8110-*napA-lysM*) group than in the LL(8110) group and the PBS group (P < 0.05) (Fig. 3). These data indicated that oral immunization with the engineered *L. lactis* expressing NapA evoked significantly elevated local and systemic humoral immune responses in mice.

### Cultivation of splenocytes and evaluation of cytokines

**Figure 4:**
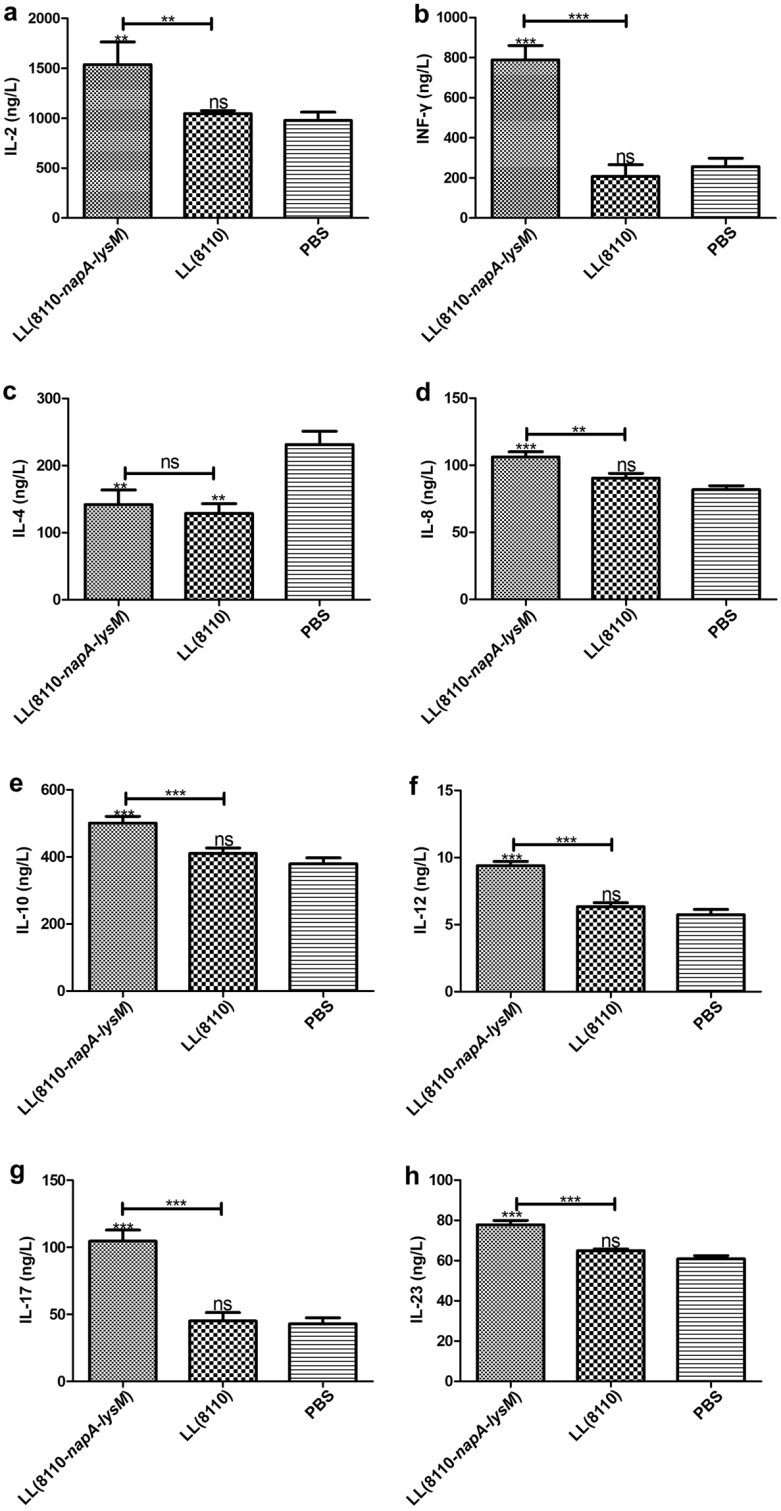
Assessment of cytokine expression levels of splenocytes. One week post immunization, splenocytes were separated from the mice and cultivated for 3 d upon irritation with *H. pylori* cell lysate antigens. The culture medium supernatant was tested for the cytokines by ELISA. ns, no significance; ^*^P < 0.05; ^**^P < 0.01; ^***^P < 0.001, compared with the PBS or designated group. This figure showed that by oral vaccination the engineered *L. lactis* producing NapA induced significantly elevated production of IL-2, IL12 and INF-γ, and reduced expression of IL-4, demonstrating the effect of the engineered strain on redirection of Th2 to Th1 response. Notably, the markedly enhanced IL-23 and IL-17 expression in the vaccinated mice demonstrated a polarized Th17 response driven by the *L. lactis*-delivered NapA.

ELISA assays of cytokines in the splenocyte culture supernatant resulted in that the group vaccinated with the engineered *L. lactis* had significantly elevated IL-2, IL-12, IL-17, IL-23 and INF-γ levels and reduced production of IL-4, compared with PBS group or LL(8110) group (Fig. 4). The IL-8 production level in the vaccinated group was significantly higher than that in the PBS group (Fig. 4d). These results demonstrated the effect of the engineered strain on redirection of Th2 to Th1 response. Notably, the markedly enhanced IL-23 and IL-17 expression in the vaccinated mice demonstrated a polarized Th17 response driven by the *L. lactis*-delivered NapA. The effect of *H. pylori* NapA on Th17 response was observed for the first time, which may greatly affect the clinical application of NapA.

### *H. pylori* challenge and gastric examination

**Figure 5:**
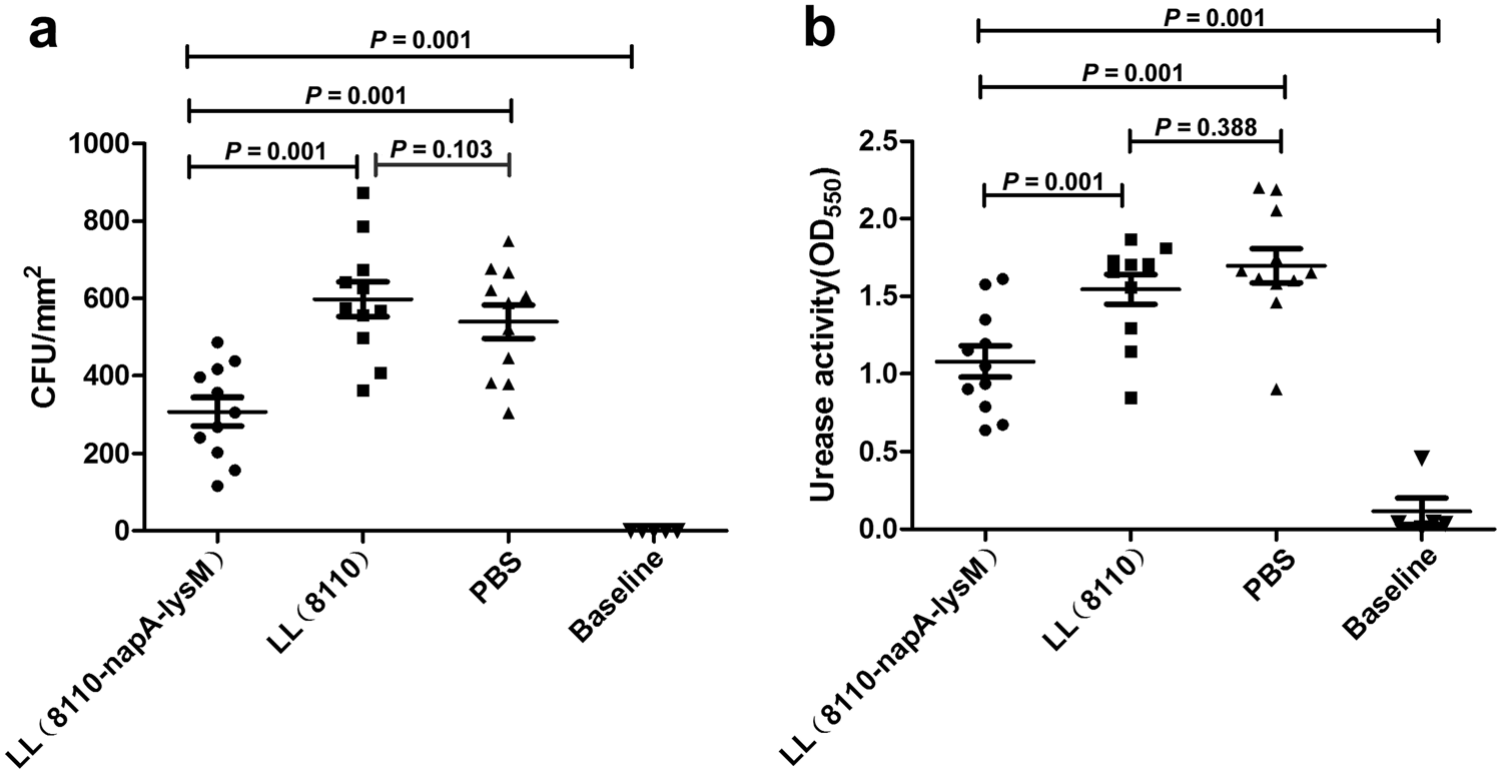
Evaluation of *H. pylori* colonization in mice by quantitative cultivation (**a**) and urease activity assays (**b**). The gastric walls of mice were sampled one week after the last challenge with *H. pylori*. The bacterial colonization was assessed by rapid urease tests and quantitative bacterial culture. ^*^P < 0.05; ^**^P < 0.01; ^***^P < 0.001, compared with the PBS or designated group. Baseline, the values of the BALB/c mice (n = 10) untreated with either *L. lactis* or *H. pylori*. The results showed that the NapA delivered by *L. lacitis* significantly reduced the bacterial load in the stomachs of the vaccinated mice.

The gastric examination by bacterial cultivation and urease activity assays showed that the gastric *H. pylori* burden and urease activity in LL(8110-*napA-lysM*) group were significantly lower than those in LL(8110) group and PBS group, but higher than those in Baseline group (n = 10) (Fig. 5). The results showed that the oral vaccination with the *L. lacitis*-delivered NapA was capable of significantly reducing the bacterial load in the stomachs of the mice challenged with *H. pylori*, but unable to protect the mice from infection.

**Figure 6:**
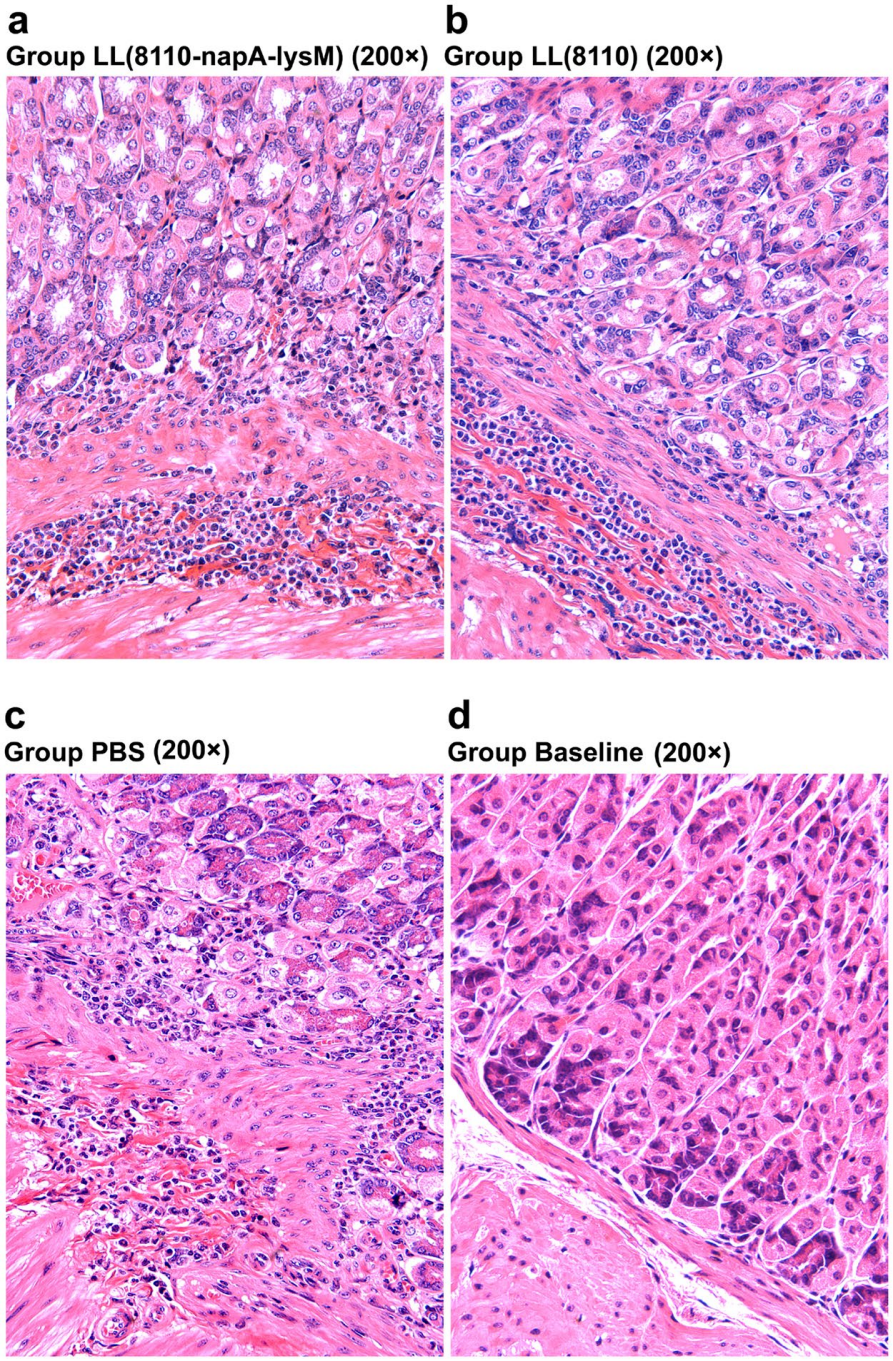
Histological observations of mouse gastric tissues following *H. pylori* challenge. The gastric slide specimens were prepared by paraffin section and hematoxylin-eosin (HE) staining. There was infiltration of lymphocytes and polymorph nuclear cells in gastric mucosa and submucosa in both the vaccinated mice (**a**) and the controls treated with *L. lactis* (pNZ8110) (**b**) or PBS (**d**–**f**). No obvious inflammation response was observed in most mice (7/8) of the Baseline group given neither immunization nor challenge (**c**).

**Figure 7:**
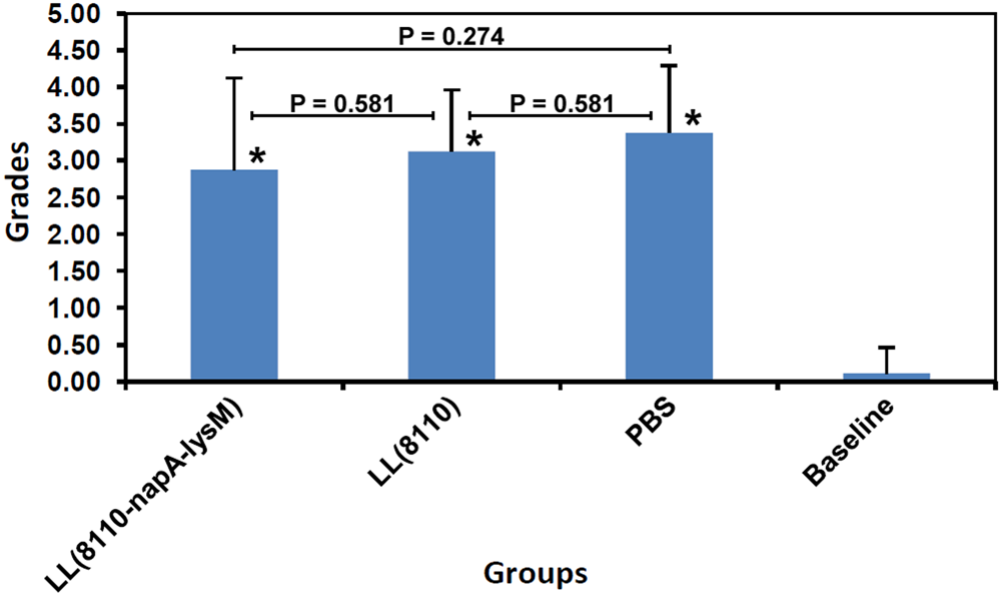
Histological grading of mouse gastric inflammatory changes. The extent of gastric inflammation was graded using the method previously reported^57^. No significant difference in the inflammatory response was detected between the immunized group (2.88 ± 1.25, n = 8) and the unimmunized groups, LL(8110) group (3.13 ± 0.83, n = 8) and PBS group (3.38 ± 0.92, n = 8) (P > 0.05). The Baseline group had significantly lower level of inflammatory changes (0.13 ± 0.35, n = 8) than all the *H. pylori-*gavaged groups (P = 0.00).

Pathological analysis was carried out to evaluate gastric inflammatory responses. Infiltration of lymphocytes and polymorph nuclear cells in gastric mucosa and submucosa were observed following the challenges in both the immunized mice and the controls administered with *L. lactis* (pNZ8110) or PBS (Fig. 6). However, no significant difference in the extent of inflammation was detected between the immunized group and the unimmunized *H. pylori*-challenged groups (Fig. 7). The Baseline group has significantly lower level of inflammatory changes than all the *H. pylori-*gavaged groups (P < 0.01). These findings indicated the oral immunization with the engineered *L. lactis* did not cause enhanced inflammation responses to *H. pylori* challenges.

## Discussion

Epidemiology studies show inverse correlation between *H. pylori* infection and frequency of asthma, which brings up a disputed issue on whether eradicating this infection is to benefit asthma patients or not^38^. In fact, it is hardly acceptable to leave *H. pylori*, as a class I carcinogen, in human body without deadline, at least for the cases with clinical manifestations. One possible solution might be using certain *H. pylori* components, like NapA, to replace the *Helicobacter* infection for depression of Th2 responses. Similarly, for other Th2 predominant allergic diseases, immunotherapies might also be established by using *H. pylori* NapA.

Among the possible administration routines, mucosal delivery of NapA should be first considered, due to the high safety, easy manipulation and rationality, especially for mucosal allergies and local cancers of digestive, respiratory and urinary tracts^39^. However, till now, no efficient production and safe utilization modes of NapA have been defined for its practical application. This study successfully produced and delivered NapA via using the safe *L. lactis* expression system, constituting a considerable step toward human use of this crucial protein.

The *L. lactis* NZ3900/pNZ8110 is a nisin-controlled expression system (NICE). The food-grade safe NZ3900 strain has been engineered carrying the genes encoding the regulators of bacterial responses to nisin concentration in the culture medium, while the plasmid vector pNZ8110 contains the nisin-inducible promoter^32^. The plasmid pNZ8110-*lysM* was constructed in our previous study, by introduction of the anchor motif *lysM* of *L. lacits acmA* gene into pNZ8110 (GenBank No. KY385375). pNZ8110-*lysM* can express heterogeneous proteins as intracellular, extracellular or surface displayed molecules, depending on which site the exogenous genes are inserted.

In this work, the *napA* gene was cloned into the pNZ8110-*lysM* at the site *Nae*I downstream of the signal peptide (SP) encoding region to make the NapA expressed in fusion with SP as a secreted protein. The termination codon of *napA* was deleted for the capablity of fusion expression with other genes. As a result of the deletion of the termination codon, a peptide of 12 amino acids encoded by the recombinant plasmid was added to the C end of the expressed NapA. Although the gene sequencing indicated that the recombinant plasmid pNZ8110-*napA-lysM* had been accurately constructed, SDS-PAGE assays showed that NapA was detectable in both the whole cell lysate and the cell wall exfoliation proteins, but not in the culture supernatant proteins. Similarly, previous studies observed that although NapA and UreB in nature are cytoplasmic proteins, they are also detectable in the outer membrane proteins of *H. pylori*^40,41^. Hence, these proteins were thought to be capable of adhering to bacterial membrane^40,41^. Another interesting finding is that the NapA expression product has a lower molecular weight than the expected (14 kD *vs* 16 kD), the mechanism underlying which needs further investigation.

Previous studies reported that production of heteroproteins in *L. lactis* might result in rather low expression levels, therefore some reports showed the expression product only by western blot assays, but not by SDS-PAGE^33,42,43^. The expressed NapA in the present study constituted 15% of the *L. lactis* cell lysate proteins, demonstrating the high expression efficiency of this engineered strain^34,42^. The westernblotting analysis confirmed that the recombinant NapA expressed in *L. lactis* had the antigenicity of its natural form. These findings suggested that the engineered strain could be a novel source of NapA.

Up till now, it remains unclear how many CFU and times of treatments are most suitable for oral vaccination with the *L. lactis*-delivered NapA. To confirm whether or not the *L. lactis* expressing NapA is capable of inducing desired immune efficacy, it is reasonable for this study to use a relatively higher dose and frequency of treatments, and the results indicated that the immunization regimens used here were effective.

In the present study, vaccination with the engineered *L. lactis* elicited markedly elevated systemic and mucosal immune responses, and thus significantly reduced bacterial colonization in the stomachs of the *H. pylori*-challenged mice. These results indicates that delivery of NapA by *L. lactis* is capable of evoking significant immune protection in mice, evidencing that *L. lactis* can be an efficient oral vaccine vehicle with adjuvant activity to induce protective immunity against *H. pylori*^33^.

To explore the mechanism underlying the immune protection and modulation, we assayed cytokine expression by splenocytes of the immunized mice. As shown, vaccination with the engineered *L. lacis* induced significantly elevated levels of IL-2, IL-12 and INF-γ, and reduced expression of IL-4, demonstrating the competence of the engineered *L. lactis* in redirection of Th2 to Th1 response. The enhanced IL-8 level in the vaccinated mice supports that the engineered strain has acquired the ability of NapA to attract neutrophils and promote inflammation. The increased IL-10 production might be due to the activation of Treg by NapA^14,44^.

Although certain previous studies indicate that *H. pylori* normally elicits an innate immune response, followed by a Th1 response, there are also evidences supporting that this bacterium can increase a Th2 or induce a mixed Th1/Th2 adaptive immune response in mice^45^. In the present study, a Th2 response was noticed in the unimmunized mice challenged with *H. pylori*, owing to the high IL-4 level in the PBS group. Our findings support existence of a Th2 or a mixed Th1/Th2 immune response to *H. pylori* challenges.

The elevated IL-23 and IL-17 expression levels demonstrated polarized Th17 responses. Since it has been evidenced that anti-*H. pylori* protective immunity is predominantly attributed to Th1 and especially Th17 responses, the immune profiles of the immunized mice herein may explain the immune protection caused by the engineered *L. lactis*^9,46^. Notably, the present study is the first report on that NapA of *H. pylori* evokes a Th17-biased response, although a previous observation demonstrated the ability of *Borrelia burgdorferi* NapA to drive a Th17 response^47^.

The exact role of Th17-biased response in tumor immunity remains unclear^48^. Some studies have documented that Th17 response is associated with reduced tumor progression, improved survival in patients with epithelial cancers, and potent antitumor efficacy in mice^49^. Others suggested that Th17 cells and related cytokines might have both antitumor and pro-tumor functions, and the links between Th17 cells and carcinogenesis are highly dependent on the context^50^. The findings of NapA’s function to up-regulate Th17 response are of significance for exploring its application.

In the past decades, incidence rate of allergic diseases, especially food allergies, have been continually increasing^51^. As reported, certain *L. lactis* isolates can prevent allergic inflammatory reactions, through endosomal recognition of *L. lactis* and its RNA by dendritic cells^36^. Engineered *L. lactis* strains producing peanut allergens were observed to modulate peanut-induced allergic immune responses in mice by redirection of Th2 to Th1 response^37^. As shown in our work, treatment with *L. lactis* NZ3900 containing the empty plasmid vector can also cause significantly reduced IL-4 production, compared with gavages with PBS. This result is in accord with the previous reports^36,37^. The IL-4 level in LL(8110-*napA-lysM*) group was significantly lower than that in the LL(8110) group, indicating the function of NapA to modulate the Th1/Th2 responses. Additionally, activation of Treg by *L. lactis* might contribute to the remarkably lowered IL-2 level in LL(8110) group, compared with the PBS group. These data demonstrate *L. lactis* as an efficient mucosal delivery vector for treating Th2-based allergic diseases^36,37^.

Presently, frequent occurrences of cancers have also become critical public health problems^52^. Biotherapies, such as bacillus Calmette-Guerin (BCG) therapy, have been used for treatment of certain cancers, and brought attractive efficacy, however, new alternative drugs are urgently needed^53^. In future, the engineered *L. lactis* expressing NapA might be applied as an immune modulatory agent, for instance, in treatment of certain immune dysregulation and carcinomas. Given that it is used in fermentation of food like milk, the lactic acid bacterium engineered here and its derivatives might have striking effect on control of these disease. The mucosal administration routine by which it takes effect is also attracted.

In the histological analysis, no significant difference was detected between the immunized group and the controls by biopsy of the gastric tissues, although the enhanced IL-8 levels in the vaccinated mice suggested the potential presence of post-immunization inflammation. On one hand, the vaccination with the engineered *L. lactis* might decrease inflammation by reducing the *H. pylori* colonization. On the other hand, NapA might induce post-immunization inflammation through polarizing Th1 immune response and elevating IL-8 expression level. The multiple functions of NapA may contribute to the histological changes observed here.

Additionally, even though *L. lactis* has been generally regarded as safe for human use, its effect on the gastro-intestinal microbiota, suspected by the administration of high doses of *L. lactis*, still needs further investigation. In future, the long-term effect of use of the engineered *L. lactis* as a potential oral vaccine candidate should also be defined. Moreover, if the immunity induced by the vaccination is incapable of lasting for enough time, the vaccination will have to be performed regularly to maintain the effect.

In summary, this work has established a novel efficient production and utilization mode of *H. pylori* NapA, demonstrated for the first time the ability of NapA to promote both Th17 and Th1 polarization, and offered an engineered *L. lactis* strain, which can be a promising anti-*H. pylori* oral vaccine candidate, a potent mucosal immune modulatory agent, and a potential antitumor strategy.

## Methods

### Bacteria and cultivation

*L. lactis* NZ3900, *H. pylori* MEL-Hp27, *H. pylori* 11637, *E. coli* TB1(pMAL-c2x*-*linker *–napA*) and plasmid pNZ8110-*lysM* were used in this work (Supplementary Table 1). *L. lactis, H. pylori* and *E. coli* were cultivated using GM17 medium, sheep blood containing Brucella agar plate and Luria Broth medium, respectively, as previously described^54,55^.

### Polymerase chain reaction of *napA*

The primers used for PCR amplification of *napA* were 5′-GCATGCCGGC ATGAAAACATTTGAAAT-3′ (Sense) and 5′-CGTGCATGCAGCTAAATGGGCTTG CAGCA-3′ (Antisense) with the endonuclease sites *Nae*I and *Sph*I underlined. Genomic DNA was isolated from *H. pylori* MEL-Hp27 using alkaline lysis method, and used as the PCR template. PCR profile included 30 cycles of 94 °C for 1 min, 50 °C for 30 s and 72 °C for 2 min.

### Construction of *L. lactis* expressing *napA*

By restriction endonuclease digestion with *Nae*I and *Sph*I (TaKaRa, China) and ligation reaction, the *napA* gene was ligated with pNZ8110-*lysM* and used for transformation of NZ3900 by electrophoration^54^. The recombinants were obtained by chloramphenicol resistance selection and PCR detection of *napA*, and identified by restriction digestion and gene sequencing^54^. The *L. lactis* strain carrying *napA* gene was referred to as *L. lactis* NZ3900 (pNZ8110-*napA-lysM*).

### Production of NapA in *L. lactis*

The engineered *L. lactis* was grown and induced for expression of NapA by adding 40 μg/L nisin to the culture at OD_600_ ≈ 0.35 and incubation for 5 h. The cellular lysate samples were prepared by centrifugation and supersonication^54^.

Samples of the culture supernatant were prepared from 50 mL of culture. The supernatant was obtained by 1 × 10^4^ rpm at 4 °C for 20 min, and filtered through 0.22 µm filter. The proteins in the filtrate were precipitated by adding trichloroacetic acid (100 mL/L), incubating at 4 °C for 16 h and centrifugation at 1 × 10^4^ rpm at 4 °C for 30 min. The pellet was resuspended in 8 mL acetone, centrifugated at 1 × 10^4^ rpm at 4 °C for 20 min, and kept at room temperature in fume hood until dry. The protein sample was added 360 μL PBS, kept at 4 °C for 3 h, and centrifugated at 1 × 10^4^ rpm 4 °C for 10 min. The supernatant was separated and used as samples of the culture supernatant.

Samples of bacterial cell wall proteins were prepared from 10 mL of culture. The cultivated bacteria were pelleted by 4.3 × 10^3^ g at 4 °C for 5 min, and washed with TES solution (50 mM Tris-HCl, 1 mM EDTA, 20% sucrose) for twice, and then suspended in 200 μL TES-LMR (30 g/L lysozyme, 0.1 g/L RNase), kept at 37 °C for 2 h with mixing at intervals for several times. The supernatant was separated by centrifugation at 2.5 × 10^4^ g 4 °C for 10 min, added ice pre-cooling trichloroacetic acid at 160 g/L, kept in ice for 20 min. The precipitate was obtained by centrifugation at 1.15 × 10^4^ g, 4 °C for 10 min, suspended in 100 μL of 50 mM NaOH solution, kept at 4 °C for 12 h, and the supernatant was separated as the cell wall protein samples.

The expression products were identified by electrophoresis and western blotting assays using mouse anti-*H. pylori* antisera as the detector antibody as reported previously^35^.

### Oral vaccination of mice and challenge with *H. pylori*

Specific pathogen free BALB/c mice, aged six weeks, were purchased from Henan Experimental Animal Center (Zhengzhou, China). The mice were assigned at random to 4 groups (Table 1). Equal amounts of male and female mice were included in every group, raised separately and housed 3~4 each cage. For LL(8110-*napA-lysM*) group (n = 22) and LL(8110) group (n = 22), the mice were treated by gavage with cell suspensions at a dose of 250 μL of *L. lactis* NZ3900 (pNZ8110-*napA-lysM*) (5 × 10^10^ CFU/mL) and NZ3900 (pNZ8110) (5 × 10^10^ CFU/mL), respectively, on day 0, 7, 14, 21, 28 and 35. For PBS group (n = 22), the mice were administered with equal volumes of PBS instead of the cell suspensions. The mice of Baseline group (n = 10) were untreated with *L. lactis*, PBS or *H. pylori*. The mice underwent fasting for 12 h and water deprivation for 4 h prior to the gavages, and were given food and drinking water 4 h after irrigation. The animal experiments have acquired approval of Institutional Review Board at Zhengzhou University, and performed under the ARRIVE guidelines.

**Table 1:** Grouping of the BALB/c mice and treatments.

Group	n	Treatments								
		0~35d	42d	42d	42d	42d	42d	42~51d	58d	58d
		Gavage with *L. lactis* or PBS 6 times	Divide into 2 Sub- groups n	EUT	Sample blood	Sample spleen cells	Sample feces	*H. pylori* challenges 4 times	EUT	Sample gastric tissues
LL(8110*- napA-lysM*)	22	√	11	√	√	√	√	×	×	×
			11	×	×	×	×	√	√	√
LL(8110)	22	√	11	√	√	√	√	×	×	×
			11	×	×	×	×	√	√	√
PBS	22	√	11	√	√	√	√	×	×	×
			11	×	×	×	×	√	√	√
Baseline	10	×	×	×	×	×	×	×	√	√

Note: √ received the treatment. × Not given the treatment. EUT euthanasia.

Seven days after the last vaccination, blood and intestinal feces samples were collected for half of the mice from each group as described below, while the remained mice were used in the *H. pylori* challenge experiment (Table 1). *H. pylori* 11637 grown on culture plate for 3 d were harvested and resuspended in culture medium to yield bacterial suspension of 1 × 10^10^ CFU/mL. The mice were fasted overnight, and received intragastric gavage with 250 μL sodium hydrogen carbonate (0.3 g/L) estimated 30 min before receiving challenges. The mice of all the 3 groups were treated by gavage with 200 μL *H. pylori* suspension each on day 7, 10, 13 and 16 post-immunization. Fasting and water deprivation were carried out in all the mice as mentioned above. The PBS group was used as a positive control group.

### Blood and intestinal feces sampling

Blood samples were fetched from orbital sinus and kept at 4 °C for 16 ~ 20 h, and then sera were separated and stored in aliquots at −20 °C. Intestinal feces was sampled following separation of the spleens (see details below), 100 mg of feces was fetched from the intestine of each mouse, and then 1 mL of PBS containing proteinase inhabitor (Phenylmethanesulfonyl fluoride, 0.1 mM) was injected by the duodenum to wash the intestinal wall. The PBS eluate was recovered and mixed with the feces. The mixture was kept at 4 °C for overnight (14~16 h). The supernatant of the mixture was separated via centrifugation at 1.2 × 10^4^ rpm for 10 min, and kept at −20 °C.

### ELISA detection of antibodies

NapA was purified by amylose affinity chromatography from IPTG-induced *E. coli* TB1 (pMAL-c2x-linker*-napA*)^56^. The specific serum IgG and fecal SIgA antibodies were quantified by ELISA using the purified NapA as the detector antigen^52^. Briefly, 96-well microplates (Beijing Solarbio, China) were coated with NapA, and the ELISA signals were developed using biotinylated goat anti-mouse IgG (Abcam, USA), goat anti-mouse SIgA (Abcam, USA) and PNPP substrate (Beijing Solarbio, China). The absorbance of each well at 450 nm (OD_450_) was read by a Microplate Reader (Tecan Sunrise, CH), and designed as indicators of the specific SIgA and IgG levels.

### Splenocytes cultivation and antigenic irritation

After blood sampling, the mice were sacrificed by spinal dislocation and soaked in 75% ethanol solution for 3 min, and then the spleens were harvested. The spleens were homogenized and passed through a 20 mesh strainer. The filtered splenocyte suspension was collected and treated with erythrocyte lysing buffer. The splenocytes were washed with D-hanks solution, and suspended to 5 × 10^6^ cells/mL in 10% calf serum containing RPMI-1640 medium. The suspension of splenocytes were added into 24-well culture plates, 400 μL each well. *H. pylori* cell lysate antigens were added at 10 μg/mL to the wells as irritation agent. The cell cultivation was performed at 37 °C, 5% CO_2_ for 72 h.

### Assessment of cytokines

The supernatant of splenocyte culture medium was separated at 72 h post cultivation by centrifugation at 3000 rpm for 20 min, and tested for quantification of cytokines interleukin (IL)-2, 4, 8, 10, 12, 17, 23 and interferon (IFN)-γ using ELISA kits (Mlbio, Shanghai, China), as instructed by the manufacturer.

### Evaluation of *H. pylori* infection

On day 7 post the challenge, the mice were fasted overnight and sacrificed by spinal dislocation. Two round pieces of the gastric wall, 4 mm in diameter, were dissected from the gastric pyloric antrum using a puncher for each mouse, homogenized on a 200 mesh strainer, and washed with 1 mL preservation fluid (containing 100 g/L sucrose and 500 mL/L fetal bovine serum). The filtrate were recovered, and used as gastric samples for urease activity assays and bacterial cultivation.

For urease activity assays, 100 μL of each gastric sample was placed in 500 μL of urea broth medium (urea 20 g/L, soy peptone 1.0 g/L, NaCl 5.0 g/L, KH_2_PO_4_ 2.0 g/L, glucose 1.0 g/L, phenol red 7.5 × 10^−2^ g/L), and kept at 37 °C for 5 h. The final values were read at 550 nm wavelength.

For quantitative bacterial culture, 100 μL of each gastric sample was plated onto Columbia agar plates with sheep blood and antibiotics, and cultured for 4 d as previously described^53^. Bacterial counts were expressed as colony forming unit (CFU) per square millimetre of gastric wall.

Additionally, ten BALB/c mice, free of the immunization and bacterial challenges, were employed for determining the base line of urease activity and *H. pylori* colonization levels of the mice.

### Gastric histological examination

Histological examination was performed for eight mice per group to evaluate gastric inflammation responses. In brief, after being sampled as mentioned above, the stomach tissue remained was fixed using 10% neutral formaldehyde solution, and checked by paraffin section and hematoxylin-eosin (HE) staining. The gastritis of the mice was graded using the method previously reported^57^.

### Statistical analysis

The data were analyzed using the software package SPSS 21.0. The measurement data were expressed as means ± standard deviation $(\overline{x}\pm s)$, and compared by one-way variance analysis. The pairwise comparisons of mean values were carried out using the least significant deviation methods (LSD). The difference was considered as significant at P < 0.05.

## Electronic supplementary material

Additional Information
