# Supplementary material for: Production and delivery of Helicobacter pylori NapA in Lactococcus lactis and its protective efficacy and immune modulatory activity
Source: Sci Rep. 2018 Apr 24;8:6435. doi: 10.1038/s41598-018-24879-x (PMC5915382; doi:10.1038/s41598-018-24879-x)
Supplement: Supplementary file 1 — Additional Information [file 41598_2018_24879_MOESM1_ESM.pdf]

Title Page

Title: Production and delivery of *Helicobacter pylori* NapA in *Lactococcus lactis* and its protective efficacy and immune modulatory activity

Authors: Xiaoyan Peng, Rongguang Zhang, Guangcai Duan, Chen Wang, Nan Sun, Linghan Zhang, Shuaiyin Chen, Qingtang Fan, Yuanlin Xi

Supplementary Table 1. Plasmid vectors and bacterial strains used herein

	**Profiles**	**Reference**
Plasmids		
pNZ8110-*lysM*	*cm*^{r}, *E. coli*-*L. lactis* shuttle vector with the usp45 signal sequence and the *nisA* promoter, constructed by introducing the anchor motif *lysM* of *L. lactis acmA* gene into the plasmid pNZ8110 (NIZO Food Research, Netherlands).	Genbank No. KY385375
pNZ8110-*napA*-*lysM*	*cm*^{r}, pNZ8110-*lysM* carrying *napA*	This study Genbank No. KY385374
pMAL-c2x-*linker*-*napA*	*amp*^{r}, pMAL-c2x (NEB, England) carrying *a linker* (5'-GGAGGCGGT-3') and *napA* gene	Previous study [52]
Strains		
L. lactis NZ3900	Derivatives of *L. lactis* subsp. cremoris MG1363, lacF$^-$, pepN::nisRnisK, food grade	NIZO Food Research, Netherlands
E. coli TB1	F-, rpsL(StrR)ara, [Φ80dlac,Δ(lacZ)M15], Δ(lac-proAB)	NEB, England
L. lactis NZ3900 (pNZ8110-*napA*-*lysM*)	*L. lactis* NZ3900 harboring pNZ8110-*napA*-*lysM, cm*^{r}	This study
E. coli TB1 (pMAL-c2x-linker-*napA*)	*E. coli* TB1 harboring pMAL-c2x-*linker -napA*, *amp*^{r}	Previous study [52]
H. pylori MEL-Hp27	*cagA+, vacA+*, isolated from a Chinese patient with chronic atrophy gastritis	CGMCC No.1338*
H. pylori 11637	*cagA+, vacA+*, type strain	NCTC11637

*CGMCC, China General Microbiological Culture Collection Center

```
ORIGIN
        1 atgaaaacat ttgaaatttt aaaacatttg caagcggatg cgatcgtgtt gtttatgaaa
       61 gtgcataact tccattggaa tgtgaaaggc acggattttt ttaatgtaca taaagctact
      121 gaagaaattt atgaagagtt tgcggacatg tttgatgatc tcgctgaaag gatcgttcaa
      181 ttaggacacc accccttagt cactttatcc gaagcgatca aactcactcg tgttaaagaa
      241 gaaactaaaa cgagcttcca ctctaaagac atttttaaag aaattctaga ggactataaa
      301 cacctagaaa aagaatttaa agagctctct aacaccgctg aaaaagaagg cgataaagtc
      361 accgtaactt atgcggatga tcaattggcc aagttgcaaa aatccatttg gatgctgcaa
      421 gcccatttag cttaa
//
```

Supplementary Figure 1. The sequence of the amplified *napA* gene of *H. pylori* MEL-Hp27. This sequence is identical to the published (GenBank No. AY366361).

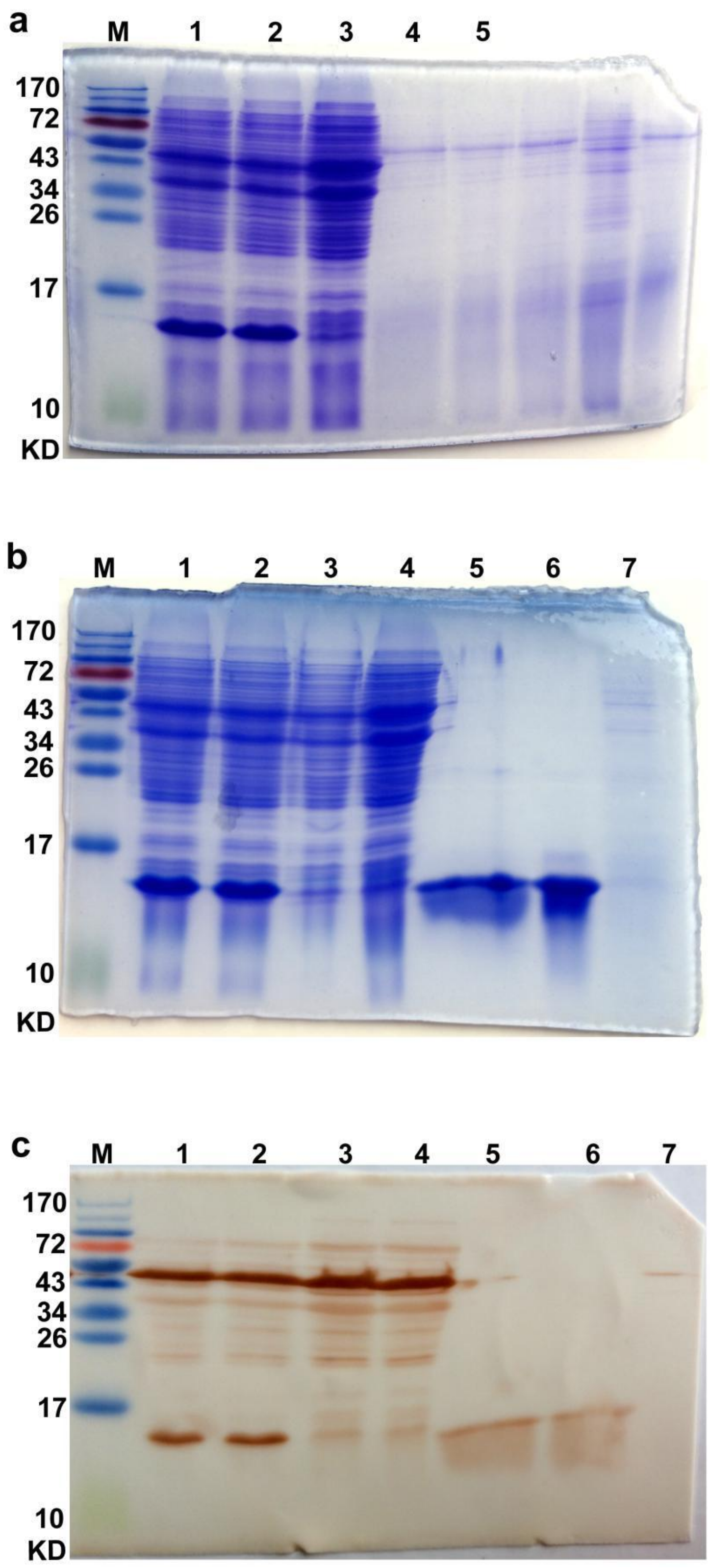

Supplementary Figure 2. Full-length gels and blots. SDS-PAGE (**a,b**) and western blotting analysis (**c**) of *L. lactis* cell lysate, cell wall and culture

supernatant proteins. The *L. lactis* strains were induced to express NapA using nisin. M, Protein markers. (**a**) Lane 1, 2**,** Cell lysates of *L. lactis* NZ3900 (pNZ8110-*napA*-*lysM*); Lane 3, Cell lysates of NZ3900 (pNZ8110-*lysM)*; Lane 4, 5, Culture supernatant of NZ3900 (pNZ8110-*napA*-*lysM*) and NZ3900 (pNZ8110-*lysM*)*,* respectively. (**b**) Lane 1, 2**,** Cell lysates of NZ3900 (pNZ8110 -*napA*-*lysM*); Lane 3, 4, Cell lysates of NZ3900 (pNZ8110-*lysM*); Lane 5, 6, Cell wall proteins of NZ3900 (pNZ8110-*napA*-*lysM*); Lane 7, Cell wall proteins of NZ3900 (pNZ8110-*lysM*). (**c**) Lane 1, 2, Cell lysates of NZ3900(pNZ8110 -*napA*-*lysM*); Lane 3,4, Cell lysates of NZ3900 (pNZ8110- *lysM*); Lane 5,6, Cell wall proteins of NZ3900 (pNZ8110-*napA*-*lysM*); Lane 7, Cell wall proteins of NZ3900 (pNZ8110-*lysM*). SDS-PAGE and westernblot assays showed that the recombinant NapA protein was detectable both in cell lysate and cell wall protein samples, and possessed immunoreactivity with mouse anti-*H. pylori* sera.
